# Chemical Profiling of *Lobelia chinensis* with High-Performance Liquid Chromatography/Quadrupole Time-of-Flight Mass Spectrometry (HPLC/Q-TOF MS) Reveals Absence of Lobeline in the Herb

**DOI:** 10.3390/molecules23123258

**Published:** 2018-12-10

**Authors:** Haixing Wang, Yuanyuan Li, Yeqing Huang, Chunyan Zhao, Hon-Yeung Cheung

**Affiliations:** 1 Department of Biomedical Sciences, City University of Hong Kong, Tat Chee Avenue, Kowloon, Hong Kong, China; wangjz84@gmail.com (H.W.); yuanyli4@unc.edu (Y.L.); crystal.15@163.com (Y.H.); chunyan0229@126.com (C.Z.); 2 Biotechnology and Health Centre, Shenzhen Research Institute, City University of Hong Kong, Shenzhen 518057, China

**Keywords:** *Lobelia chinensis*, *Lobelia inflata*, lobeline, liquid chromatography, mass spectrometry, chemical profiling

## Abstract

*Lobelia chinensis* is a kind of herbal medicine widely distributed and used in Asia. The chemical components of this herb, however, have not been well studied until now. Lobeline, as an essential and famous bioactive compound in *Lobelia* genus, has been assumed to be present in *L. chinensis*. In order to ascertain its presence and, more importantly, proper use of this herb, chemical profiling this herb with highly sensitive and high-resolution analytical mass spectrometry was applied. In this study, high-performance liquid chromatography coupled with quadrupole time-of-flight mass spectrometry (HPLC/Q-TOF MS) method was employed to systematically profile the chemical constituents of *L. chinensis* for the first time. Comparative chemical profiling study of *L. chinensis* and *Lobelia inflata* was also conducted to provide evidence whether lobeline is present or not. Piperidine alkaloids except for lobeline, alkaloid-lignan hybrids, flavonoids, polyacetylenes, nonanedioic acid, and some new phytochemicals were successfully identified in *L. chinensis* simultaneously. Comparing to the chemical profiles of *L. inflata*, lobeline was found to be absent in *L. chinensis*. All of the secondary metabolites in *L. chinensis* were determined with the HPLC/Q-TOF MS method. The absence of lobeline in *L. chinensis* was confirmed after this extensive study.

## 1. Introduction

*Lobelia chinensis*, also named as the Chinese Lobelia, is a plant belonging to the genus of *Lobelia* in Campanulaceae family. This plant is mainly distributed in East Asia, especially in China. The dried plant has been used as an herbal medicine by for long time [1]. In the famous traditional Chinese medicine book, namely, ‘Compendium of Matera Medica,’ *L. chinensis* was reported to promote urination, reduce edema, cleanse heat and toxicants. Traditionally, this herb is used to treat snakebites and to relieve edema according to the practices of Chinese folk medicines. This plant is included in the contemporary Chinese Pharmacopoeia and officially regarded as an herbal medicine [2].

Modern pharmacological study of *L. chinensis* revealed that this herb possesses some biological activities on cancer, bacteria, and viruses [3]. It is also reported to have inflammation modulatory activities [4]. In order to demonstrate which bioactive metabolites in this plant are responsible for these activities, some flavonoids, polyacetylenes, and alkaloids, including two pyrrolidine alkaloids and seven piperidine alkaloids in this herb have been isolated and documented in the literature [5,6,7,8]. However, the presence of lobeline, which is a crucial pyrrolidine alkaloid, has not been scientifically established, unlike another well-known *Lobelia* plant, namely *Lobelia inflata* which is native to North America, of which lobeline has been isolated and identified for the first time. This pyrrolidine alkaloid has been found to interact with neurotransmitter transporters, including serotonin transporter, dopamine transporter and vesicular monoamine transporter 2, or act as an agonist or antagonist at nicotinic acetylcholine receptors [9,10]. Due to morphological similarities between *L. inflata* and *L. chinensis*, lobeline was frequently assumed to be present in the latter as well [1,4]. However, there is no direct evidence to prove its presence in *L. chinensis* so far. Hence, some in-depth exploration of lobeline in *L. chinensis* is necessary.

As a powerful analytical tool, high-performance liquid chromatography/quadrupole time-of-flight mass spectrometry (HPLC/Q-TOF MS) has been used for characterization of many complex materials [5,10,11,12,13]. The high sensitivity and high accuracy features of this technique provide detailed chemical profiles for identification of different chemical components in complex materials, like herbal substances, and is much more accessible and feasible. The acquired accurate mass spectra of elemental composition, combined with tandem mass spectrometry (MS/MS) spectra allow the individual chemical structures to be detected and identified.

In this report, HPLC/Q-TOF MS in both positive and negative ionization modes was employed to characterize the chemical constituents in *L. chinensis* for the first time. Investigation of lobeline in *L. chinensis* was conducted through HPLC/Q-TOF MS based chemical profiling of 10 batches of *L. chinensis* samples from different regions of China and one batch of *L. inflata* sample collected from North America. It was concluded that lobeline is absent in the Chinese Lobelia.

## 2. Materials and Methods

### 2.1. Plant Materials

**Figure 1:**
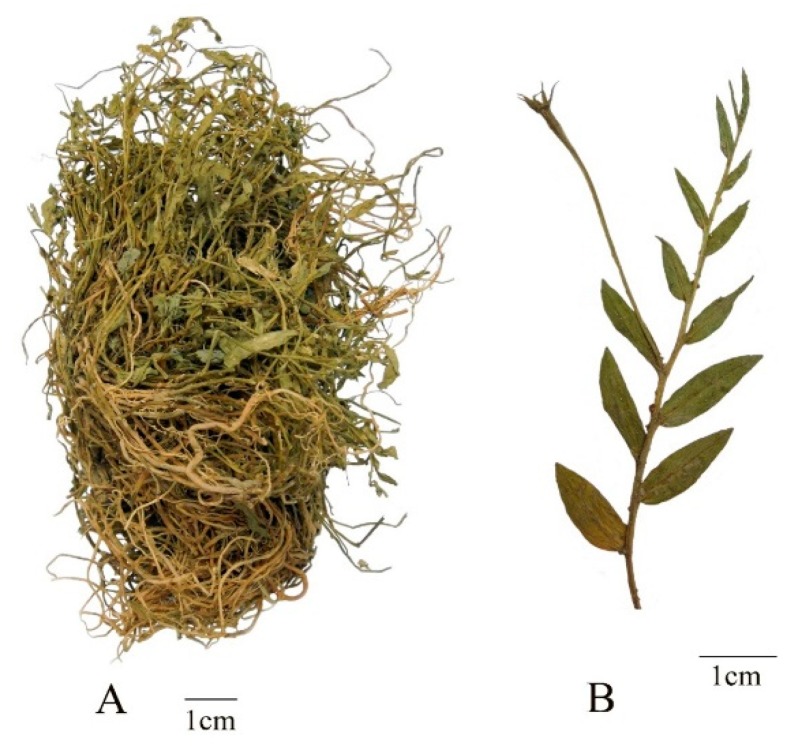
The representative photo of the collected herbal sample (**A**) and the herbarium specimen (**B**) of *L. chinensis*.

Ten batches of *L. chinensis* samples (LC-01, LC-02, …, LC-10), as shown in Table 1, were collected from different areas of China in the summer. The representative picture of the collected herbal sample and herbarium specimen of *L. chinensis* were shown in Figure 1A,B respectively. A batch of *L. inflata* was purchased from the supermarket in the United States. All these plant materials were authenticated by Professor Min Li from Chengdu University of Traditional Chinese Medicine based on their botanical features.

### 2.2. Chemicals and Solvents

Methanol and acetonitrile were of gradient grade for HPLC (Merck, Darmstadt, Germany). Formic acid (FA) was purchased from Scharlau (Spain). Water was purified with Millipore Mill-Q Gradient A10 equipment (MA, USA). Diosmin, linarin, lobetyolinin and lobetylolin with purity higher than 98% (HPLC) were purchased from Baoji Herbest Bio-Tech Co., Ltd. (Shanxi, China). Lobeline hydrochloride was purchased from Sigma-Aldrich (St. Louis, MO, USA).

### 2.3. Sample Extraction

All dried plant materials were pulverized and pass through a 50-mesh sieve prior to being extracted. Ten grams of sample powder was extracted with 20 mL methanol by sonication for 20 min and subjected to centrifugation at 4500 r.p.m. for 5 min. After centrifugation, the sample solution was collected and filtered with a 0.22 µm nylon syringe filter (Xiboshi, Tianjin, China) and the filtrate was used for subsequent analysis.

### 2.4. HPLC/Q-TOF MS Conditions

An Agilent 1200 series HPLC system (Santa Clara, CA, USA) consisting of a degasser, binary gradient pump, autosampler, and column thermostat was coupled with a Q-TOF mass spectrometer (QSTAR Elite, Applied Biosystems/MDS Sciex) equipped with a standard electrospray ionization ion source was used. High performance liquid chromatographic separation was performed on a Waters XBridge C18 reversed-phase column (2.1 × 100 mm, 3.5 µm) at a column temperature of 40 °C. Gradient elution was conducted with water (0.1% formic acid) (A) and acetonitrile (0.1% formic acid) (B): 0 min, 95% A; 5 min, 95% A; 25 min, 72%, and 40 min, 72% A. The solvent flow rate was 300 µL∙min^−1^ and the injection volume was 5 µL.

Mass spectrometry data was acquired in both positive and negative ionization modes with a mass range of 50–1000 Dalton. Instrumental parameters were optimized as follows: Voltage, 5500 V (positive) and −4500 V (negative); source gas 1 (nebulizer gas), 35 psi; source gas 2 (auxiliary gas), 60 psi; curtain gas, 30 psi; Turbo gas temperature, 500 °C; declustering potential, 80 V (positive) and −80 V (negative); focusing potential, 380 V (positive) and −380 V (negative); declustering potential 2, 10 V (positive) and −10 V (negative); collision gas, 3 psi. Nitrogen was used in all cases. For MS/MS analysis, the most abundantly charged ions from 150 to 700 above 100 counts threshold were selected for collision-induced dissociation (CID) using the information-dependent acquisition (IDA) method with automatic collision energy. This MS/MS analysis was conducted for each sample during HPLC/Q-TOF MS analysis. The instrument was calibrated using a solution of sodium bromate in both positive and negative modes prior to data collection. For accurate mass measurements, the solution of leucine-enkephalin ([M + H]^+^ 556.2771 and [M − H]^−^ 554.2615) at a concentration of 0.5 µM was used for internal calibration in both positive and negative ionization modes. The calibration solution was continuously spiked into post-column sample solution using a PEEK tee fitting (0.020”, Vertical, Thailand) by a syringe pump (Harvard Apparatus 11 Plus, Holliston, MA, USA) at a flow rate of 5 µL∙min^−1^ during full analysis. An Analyst QS 2.0 software was employed for data acquiring and processing.

## 3. Results and Discussion

### 3.1. HPLC/Q-TOF MS Analysis of the Chemical Constituents of L. Chinensis

**Figure 2:**
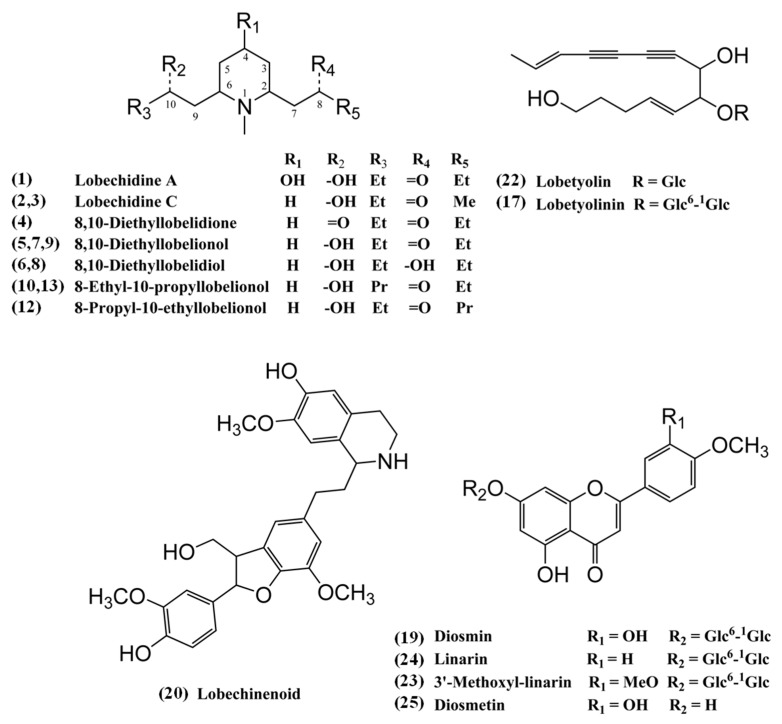
Chemical structures of the identified chemical constituents from *L. chinensis*.

**Figure 3:**
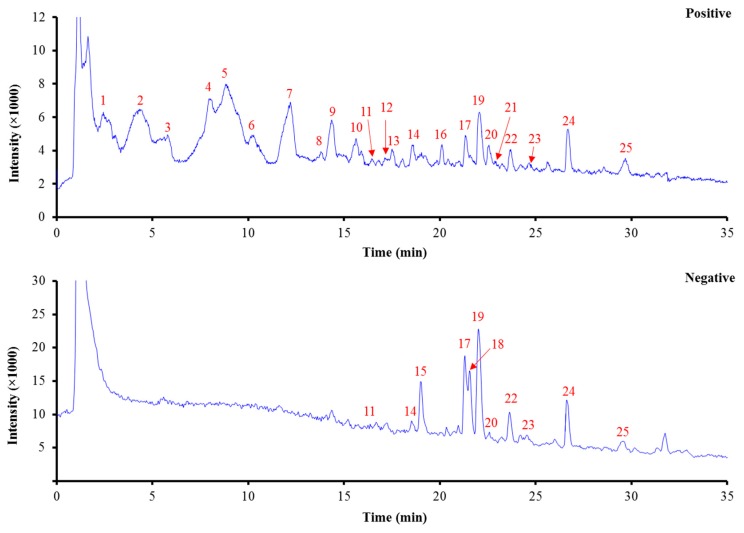
Total ion current (TIC) chromatograms of *L. chinensis* in both positive and negative ionization modes.

Being a powerful tool for analysis of secondary plant metabolites, an HPLC system coupled with a high-resolution Q-TOF mass spectrometer was employed for the chemical profiling the *L. chinensis* (LC-01) extract. Based on the required retention time, accurate masses and MS/MS spectra, and comparison of these data with previous references or available chemical standards, individual chemical components (Figure 2) in the total ion current (TIC) chromatograms of *L. chinensis* were assigned and listed as shown in Table 2 and Figure 3.

In the TIC chromatogram of *L. chinensis* in positive ionization mode, Peaks 1−10, 12 and 13 were assigned as piperidine alkaloids. According to the reported fragmentation behaviors of 2,6-disubstituted *N*-methylpiperidine alkaloids where cleaving α to N is preferred, the cleavage of one side chain, or both side chains with hydrogen transfer to leave singly or doubly unsaturated *N*-methylpiperidine, or the cleavage of *N*-methyl group is the characteristic behavior of 2,6-disubstituted *N*-methylpiperidine alkaloids in their MS/MS spectra [10,21,22].

Peak 1, which provided protonated molecular ion [M + H]^+^ at *m*/*z* 258.2052, was identified as a stereoisomer of lobechidine A which was first isolated and determined from *L. chinensis* by Yang et al. in 2014 [8]. In the subsequent MS/MS experiment, *m*/*z* 184 came from the neutral loss of side chain ethyl-2-hydroxyethyl unit (C_4_H_10_O), *m*/*z* 240, 168 and 150 were from the successive loss of one water molecule, the other side chain ethyl-2-ketoethyl moiety (C_4_H_8_O) and another water molecule, *m*/*z* 96 and 94, which were 2 Da less than the characteristic singly (*m*/*z* 98) and doubly (*m*/*z* 96) unsaturated *N*-methlypiperidine, and indicated the presence of a 3, 4-double bond induced by the elimination of the 3-hydroxoyl group in this 2,6-disubstitued *N*-methylpiperidine alkaloid.

Peaks 2 and 3, which had [M + H]^+^ at *m*/*z* 228.1965 and 228.1962, respectively, were assigned as two stereoisomers of the reported lobechidine C [8]. These two stereoisomers showed very similar MS/MS spectra. Fragments *m*/*z* 210 and 154 indicated the loss of a water moiety and an ethyl-2-hydroxyethyl side chain respectively, successive losses of the other methyl-2-ketoethyl side chain (C_3_H_6_O), a water moiety and an *N*-methyl group finally led to fragmentation ions at *m*/*z* 170, 152 and 138, and *m*/*z* 156 came from the losses of the methyl-2-ketoethyl side chain and *N*-methyl group. Typical fragments for this 2,6-disubstituted *N*-methylpiperidine alkaloid were detected at *m*/*z* 96 and 98 also.

Peak 4 providing [M + H]^+^ at *m*/*z* 240.1951 was assumed to be 8,10-diethyllobelidione with two identical ketonic side chains regarding the MS/MS fragments at *m*/*z* 168 and 96 from successive losses of one and the other ethyl-2-ketoethyl side chain [14]. Peaks 5 and 7, giving [M + H]^+^ at *m*/*z* 242.2132 and 242.2106, respectively, were identified as two stereoisomers of 8,10-diethyllobelionol [15]. Almost the same fragmentation ions could be obtained as given in Table 2. These fragmentation ions appeared at *m*/*z* 224 from loss of a water moiety, *m*/*z* 168 from loss of side chain ethyl-2-hydroxyethyl moiety, *m*/*z* 170 and 152 from successive cleavages of an ethyl-2-ketoethyl unit and a water molecule. The characteristic ion pair at *m*/*z* 96 and 98 was always detected in the MS/MS spectra. Peak 9 provided an accurate mass of [M + H]^+^ at *m*/*z* 242.2098, the same as Peaks 5 and 7. However, the obtained MS/MS spectrum predominated by fragments at *m*/*z* 58 and 116 showed different patterns to Peaks 5 and 7. These dominant fragments could only be detected with very low abundance in the MS/MS spectra of Peaks 5 and 7. Therefore, Peak 9 was also tentatively identified as another stereoisomer of 8,10-diethylobelionol.

Peaks 6 and 8 had similar MS/MS spectra and gave [M + H]^+^ at *m*/*z* 244.2261 and 244.2265 respectively, which were 2 Da upward shifted from the protonated molecular ion of 8,10-diethylobelionol. The same MS/MS fragments as Peaks 5 and 7 at *m*/*z* 170, 152 and 98 together with a new fragmentation ion at *m*/*z* 226 from the loss of a water molecule were acquired finally. According to the fragmentation behaviors of 2,6-disubstituted *N*-methylpiperidine alkaloids, Peaks 6 and 8 were identified as two stereoisomers of 8,10-diethyllobelidiol [16].

Peaks 10, 12 and 13 provided [M + H]^+^ at *m*/*z* 256.2269, 256.2265 and 256.2272, respectively. As shown in Table 2, the MS/MS spectra of Peaks 10 and 13 were almost identical which were different from Peak 12. The protonated molecular ions and fragmentation ions at *m*/*z* 166, 184 and 238 showed an obvious 14 Da (CH_2_) upward shift comparing with 8,10-diethyllobelionol. The characteristic ion pair at *m*/*z* 98 and 96 from the elimination of both side chains and fragments at *m*/*z* 238 from the loss of a water unit were detected in all MS/MS spectra. For Peaks 10 and 13, fragments at *m*/*z* 184 and 166 indicated the successive loss of an ethyl-2-ketoethyl side chain and a water molecule, and fragments at *m*/*z* 168 indicated the loss of a new propyl-2-hydroxyethyl side chain (C_5_H_12_O). Regarding the described fragmentation patterns of 2,6-disubstituted *N*-methylpiperidine alkaloids, Peaks 10 and 13 were assumed to be two stereoisomers of 8-ethyl-10-propayllobelionol where the 10-ethyl group in 8,10-diethyllobelionol was substituted with a propyl group. Peak 12 was identified as a stereoisomer of 8-propyl-10-ethyllobelionol because of fragments at *m*/*z* 182 from the loss of ethyl-2-hydroxyethyl moiety, *m*/*z* 170 and 152 from successive losses of a propyl-2-ketoethyl side chain (C_5_H_10_O) and a water molecule. This was the first time for the identification of new 8-ethyl-10-propayllobelionol and 8-propyl-10-ethyllobelionol as secondary metabolites in *L. chinensis*.

Peaks 11, 14, 16 and 20 produced similar MS/MS spectra. The same fragmentation ions at *m*/*z* 137, 265, 285, 297, 311, 323 and 341 were detected in their MS/MS spectra. In the beginning, only Peak 20 providing *m*/*z* at 508.2336 was assumed to be a stereoisomer of alkaloid-lignan hybrid lobechinenoid according to the accurate mass measurement result. This compound was first isolated as a mixture of two diastereomeric pairs of enantiomers lobechinenoids A-D from the aerial parts of *L. chinensis* [18]. The fragment at *m*/*z* 478 indicated the loss of a methyloxyl group. Peak 16 at *m*/*z* 494.2177, which was 14 Da (CH_2_) less than the protonated molecular ion of lobechinenoid, was tentatively identified to be a stereoisomer of demethyllobechinenoid for the first time. Peak 11 at *m*/*z* 656.2699 and Peak 14 at *m*/*z* 670.2863 were 162 Da (C_6_H_10_O_5_) more than the protonated molecular ions of demethyllobechinenoid and lobechinenoid respectively, which indicated the addition of a glucose molecule. Therefore, Peaks 11 and 14 were tentatively identified as lobechinenoid glucoside and demethyllobechinenoid glucoside respectively. These two glycosides were not reported in previous research [4,5,6,8,14,15,16,18]. Therefore, this was also the first detection of these two glycosides in *L. chinensis*.

Peak 15, producing ion at *m*/*z* 494.2040, was only detected in negative ionization mode with high abundance and could not be identified currently. Peaks 17 and 22, which could be detected in both positive and negative ionization modes, were identified as polyacetylenes comparing the retention time, accurate masses and MS/MS spectra with reference standards. Peak 17, yielding protonated molecular ion [M + H]^+^ at *m*/*z* 559.2396 and deprotonated molecular ion [M − H]^−^ at *m*/*z* 557.2223, was identified as lobetyolinin. The mass spectrum was dominated by [M + NH_4_]^+^ or [M + Cl]^−^ and [M + HCOO]^−^ in positive or negative ionization mode. In MS/MS spectrum of [M + NH_4_]^+^, successive losses of two glucose moieties led to fragments at *m*/*z* 397 and 217 respectively. Peak 22 gave [M + H]^+^, [M + NH_4_]^+^, [M − H]^−^, [M + Cl]^−^ and [M + HCOO]^−^, all of which were 162 Da downward shifted from corresponding ions of lobetyolinin. Therefore, Peak 22 was identified as lobetyolin, a glucose molecule eliminated metabolite of lobetyolinin. The fragment at *m*/*z* 217 from loss of the remaining glucose group was detected in MS/MS spectrum. Both lobetyolinin and lobetyolin had been reported as chemical components of *L. chinensis* previously [4,5,6]. The unidentified Peak 15 showed similar fragments as lobetyolinin and lobetyolin at *m*/*z* 59, 71, 89, 113 and 119, so it was suspected as an analogue of lobetyolinin and lobetyolin which was not reported before.

Peak 18, giving [M − H]^−^ at *m*/*z* 187.0957, was identified as nonanedioic acid according to its MS/MS spectrum and accurate mass [17]. Peaks 19 and 24 were identified as flavonoids linarin and diosmin respectively according to reference standards and previous reports [4,5]. Peak 19 showed [M + H]^+^ at *m*/*z* 609.1812 and [M − H]^−^ at *m*/*z* 607.1662. In positive ionization mode, fragments at *m*/*z* 463 and 301 were induced by successive losses of two glucose units. In negative ionization mode, fragments at *m*/*z* 299 and 284 were derived from successive losses of the sugar chain and methyl (CH_3_) group. The pseudo-molecular ions ([M + H]^+^ and [M − H]^−^) and some MS/MS fragments of diosmin from Peak 24 appeared to be a 16 Da downward shift from corresponding ions of Peak 19. For Peak 23, [M + H]^+^ at *m*/*z* 623.1986 and its fragments at *m*/*z* 315 and 477 were 30 Da (CH_2_O) more than corresponding ions of linarin. Referring to the chemical structures of previously detected flavonoids, Peak 23 was proposed as 3’-methoxyl-linarin which was detected in the leaves of Abies pindrow before [19]. This is the first time that this compound was found in *L. chinensis*.

Peak 21, producing [M + H]^+^ at *m*/*z* 679.5180, was unidentified. The MS/MS fragments were listed in Table 2. Peak 25, giving [M + H]^+^ and [M − H]^−^ at *m*/*z* 301.0708 and 299.0560, respectively, was identified as diosmetin, which was detected as the sugar chain eliminated metabolite of diosmin [20].

To summarize, piperidine alkaloids, alkaloid-lignan hybrids, involving their corresponding glucosides, flavonoids, polyacetylenes, and one organic acid were identified from *L. chinensis*. Phytochemicals, 8-ethyl-10-propyllobelionol, 8-propyl-10-ethyllobelionol, demethyllobechinenoid, together with two alkaloid glucosides, lobechinenoid glucoside, and demethyllobechinenoid glucoside, were identified as new compounds in this study. A flavonoid compound, 3’-methoxyl-linarin, was detected in *L. chinensis* for the first time. The bioactivities of some of the detected phytochemicals were studied and demonstrated in previous research. *L. chinensis* alkaloids were testified to have anti-growth activity on cancer cells [23,24]. The detected diosmin and its aglycone form diosmetin were proved as natural dietary agonists of the aryl hydrocarbon receptor (AhR) causing a strong increase in cytochrome P450 1A1 transcription and activity [25]. Flavonoid compound linarin was evaluated as a better anti-inflammatory agent in mice and rats comparing with pectolinarin [26].

### 3.2. Investigation of Lobeline in L. Chinensis

As the most significant kind of piperidine alkaloid in the *Lobelia* genus, especially in *L. inflata*, lobeline showed remarkable biological activities on the central nervous system (CNS) [9,27]. Lobeline was often regarded as the bioactive secondary metabolite of *L. chinensis* also [1,4]. However, in our study on the chemical profiling of *L. chinensis* using sensitive HPLC/Q-TOF MS, twelve stereochemically different piperidine alkaloids were detected without well-known lobeline. To investigate the presence of lobeline, 10 batches of *L. chinensis* together with one batch of *L. inflata* were simultaneously analyzed with HPLC/Q-TOF MS.

**Figure 4:**
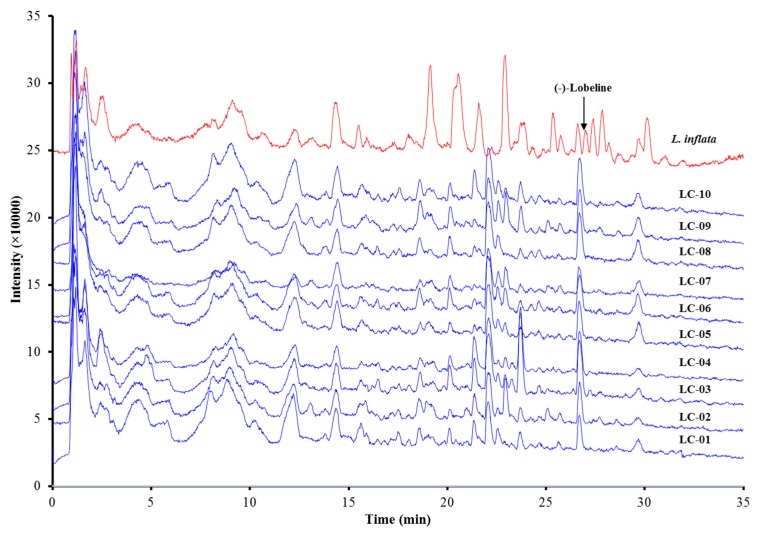
TIC chromatograms of 10 batches of *L. chinensis* (LC 01-10) and one batch of *L. inflata* in positive ionization mode.

**Figure 5:**
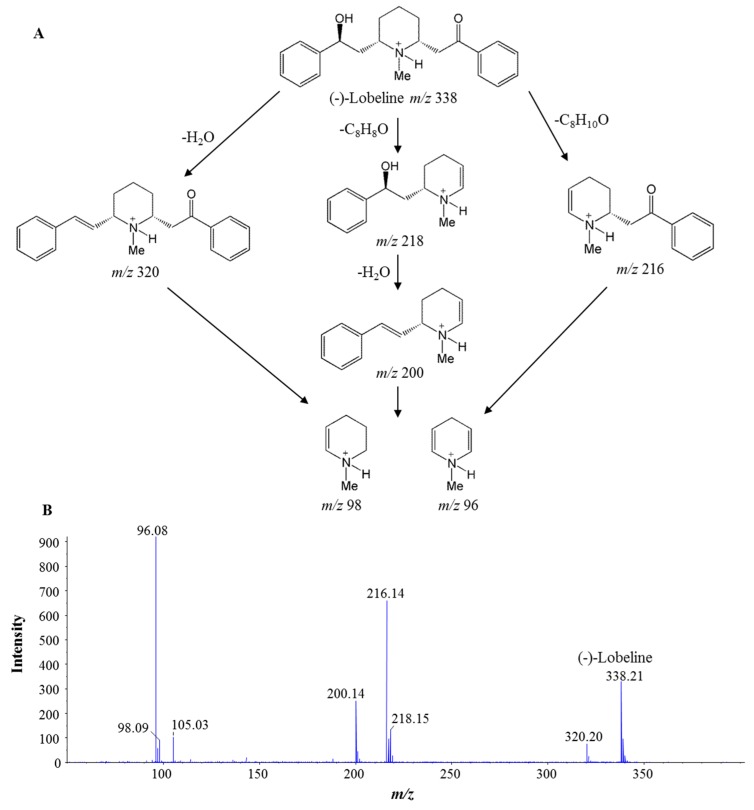
The fragmentation rules (**A**) and tandem mass spectrometry (MS/MS) spectrum (**B**) of lobeline.

The final results are shown in Figure 4. The 10 batches of *L. chinensis* samples tested showed very similar TIC chromatograms which were totally different from *L. inflata*. Lobeline was easily identified in the TIC chromatogram of *L. inflata* with a retention time at 27.0 min by the retention time and MS/MS spectrum of the reference standard and previous reports [10,22]. The fragmentation behavior and corresponding MS/MS spectrum of lobeline are shown in Figure 5. In MS/MS spectrum, neutral loss of a side chain induced the fragments at *m*/*z* 216 or 218, and successive losses of one water moiety and the ketone side chain leaded to fragments at *m*/*z* 320 and 200 respectively. Two characteristic fragments at *m*/*z* 98 and 96 belonging to unsaturated *N*-methlypiperidines were also obtained. However, in the TIC chromatograms of all 10 batches of *L. chinensis* samples collected from different regions of China, no peak belonging to (−)-lobeline with a retention time at 27.0 min could be detected. This result demonstrated that lobeline was absent in the *Lobelia* plant *L. chinensis*.

## 4. Conclusions

In conclusion, as a powerful tool for rapid identification of chemical constituents in complex materials, such as herbs, HPLC/Q-TOF MS was applied for the determination of the secondary metabolites of *L. chinensis* in this study. By comparing the acquired retention time, accurate masses and MS/MS spectra with reference standards and previous references, 12 stereochemically different piperidine alkaloids, four alkaloid-lignan hybrid analogues, four flavonoids, two polyacetylenes, and an organic acid nonanedioic acid were simultaneously discovered in *L. chinensis*. Among these phytochemicals, two types of piperidine alkaloids, 8-ethyl-10-propyllobelionol and 8-propyl-10-ethyllobelionol, and three piperidine alkaloid analogues, demethyllobechinenoid, demethyllobechinenoid glucoside, and lobechinenoid glucoside, were detected for the first time. As the most significant piperidine alkaloids detected in *Lobelia* plant *L. inflata*, lobeline was found to be absent in all 10 batches of the *L. chinensis* collected from different regions of China. This is the first systematic study on the chemical constituents of *L. chinensis* using sensitive HPLC/Q-TOF MS. The comparative investigation of *L. chinensis* and *L. inflata* provided direct and solid evidence to prove the absence of lobeline in *L. chinensis*. This research will be helpful for the quality control and further study of *L. chinensis* as a valuable herbal medicine.

## Figures and Tables

**Table 1:** Ten batches of *Lobelia chinensis* samples collected from different areas of China.

Sample Number	Origin
**LC-01**	Zhuzhou, Huan
**LC-02**	Zhuzhou, Huan
**LC-03**	Jiujiang, Jiangxi
**LC-04**	Qingyuan, Guangdong
**LC-05**	Yizhou, Guangxi
**LC-06**	Yizhou, Guangxi
**LC-07**	Chengdu, Sichuan
**LC-08**	Chengdu, Sichuan
**LC-09**	Lianqiao, Hunan
**LC-10**	Chengdu, Sichuan

**Table 2:** Chemical constituents identified from *L. chinensis* with the high-performance liquid chromatography coupled with quadrupole time-of-flight mass spectrometry (HPLC/Q-ToF MS) method in both positive and negative ionization modes.

Peak No.	t_R_ (min)	Identification	Chemical Formula	Positive Ionization Mode			Negative Ionization Mode			Ref.
				*m/z*	MS/MS Fragments	Error (ppm)	*m/z*	MS/MS Fragments	Error (ppm)	
**1**	2.5	lobechidine A	C_14_H_27_NO_3_	[M + H]^+^ 258.2052	58.1, 94.1, 96.1, 150.1, 168.1, 184.1, 240.2	−4.6	−	−	−	[8]
**2**	4.3	lobechidine C	C_13_H_25_NO_2_	[M + H]^+^ 228.1965	96.1, 98.1, 138.1, 152.1, 156.1, 170.2, 210.2	0.8	−	−	−	[8]
**3**	5.4	lobechidine C	C_13_H_25_NO_2_	[M + H]^+^ 228.1962	96.1, 98.1, 152.1, 154.1, 156.1, 170.2, 210.2	1.6	−	−	−	[8]
**4**	8.1	8,10−diethyllobelidione	C_14_H_25_NO_2_	[M + H]^+^ 240.1951	58.1, 96.1, 116.1, 168.1	−3.0	−	−	−	[14]
**5**	8.8	8,10−dietheyllobelionol	C_14_H_27_NO_2_	[M + H]^+^ 242.2132	96.1, 98.1, 152.1, 170.2, 224.2	7.1	−	−	−	[15]
**6**	10.2	8,10−diethyllobelidiol	C_14_H_29_NO_2_	[M + H]^+^ 244.2261	81.1, 98.1, 152.1, 154.1, 170.1, 226.2	−4.2	−	−	−	[16]
**7**	12.1	8,10−dietheyllobelionol	C_14_H_27_NO_2_	[M + H]^+^ 242.2106	96.1, 98.1, 152.1, 168.1, 170.2, 224.2	−3.7	−	−	−	[15]
**8**	13.8	8,10−diethyllobelidiol	C_14_H_29_NO_2_	[M + H]^+^ 244.2265	98.1, 152.1, 154.2, 170.2, 226.2	−2.5	−	−	−	[16]
**9**	14.3	8,10−dietheyllobelionol	C_14_H_27_NO_2_	[M + H]^+^ 242.2098	58.1, 116.1	−6.7	−	−	−	[15]
**10**	15.6	8−ethyl−10−propyllobelionol	C_15_H_29_NO_2_	[M + H]^+^ 256.2269	96.1, 98.1, 166.2, 184.2, 238.2	−0.7	−	−	−	−
**11**	16.4	demethyllobechinenoid glucoside	C_34_H_41_NO_12_	[M + H]^+^ 656.2699	137.1, 166.1, 175.1, 265.1, 285.1, 297.1, 311.1, 315.1, 323.1, 341.1, 464.2	−0.4	[M − H]^−^ 654.2552	−	1.1	−
**12**	17.1	8−propyl−10−ethyllobelionol	C_15_H_29_NO_2_	[M + H]^+^ 256.2265	96.1, 98.1, 152.1, 170.2, 182.2, 238.2	−2.2	−	−	−	−
**13**	17.5	8−ethyl−10−propyllobelionol	C_15_H_29_NO_2_	[M + H]^+^ 256.2272	96.1, 98.1, 166.2, 168.1, 184.2, 238.2	0.4	−	−	−	−
**14**	18.5	lobechinenoid glucoside	C_35_H_43_NO_12_	[M + H]^+^ 670.2863	137.1, 175.1, 180.1, 265.1, 285.1, 297.1, 311.1, 315.1, 323.1, 341.1, 478.2	0.7	[M − H]^−^ 668.2691	−	−1.6	−
**15**	19.0	unknown	−	−	−	−	401.1744	59.0, 71.0, 89.0, 101.0, 113.0, 119.0, 123.1, 159.0, 177.1, 221.1	−	−
**16**	20.0	demethyllobechinenoid	C_28_H_31_NO_7_	[M + H]^+^ 494.2177	137.1, 166.1, 175.1, 265.1, 285.1, 297.1, 311.1, 315.1, 323.1, 341.1, 464.2	0.8	[M − H]^−^ 492.2040	−	4.8	−
**17**	21.3	lobetyolinin	C_26_H_38_O_13_	[M + H]^+^ 559.2396 * [M + NH_4_]^+^ 576.2637 *	155.1, 199.1, 217.1, 397.2	1.9	[M − H]^−^ 557.2223 [M + Cl]^−^ 593.1975 [M + HCOO]^−^ 603.2279 *	59.0, 71.0, 89.0, 101.0, 113.0, 119.0, 143.0, 161.1, 179.1, 221.1	−1.0	[4,5,6]
**18**	21.5	nonanedioic acid	C_9_H_16_O_4_	−	−	−	[M − H]^−^ 187.0957	97.1, 125.1, 126.1, 169.1	−4.4	[17]
**19**	22.0	diosmin	C_28_H_32_O_15_	[M + H]^+^ 609.1812	301.1, 463.1	−0.3	[M − H]^−^ 607.1662	284.0, 299.1	0.7	[4,5]
**20**	22.5	lobechinenoid	C_29_H_33_NO_7_	[M + H]^+^ 508.2336	137.1, 151.1, 180.1, 265.1, 285.1, 297.1, 311.1, 315.1, 323.1, 341.2, 478.2	1.2	[M − H]^−^ 506.2180	−	1.3	[18]
**21**	22.9	unknown	−	679.5180	100.1, 182.2, 209.2, 226.2, 326.3, 336.2, 435.3, 452.4, 661.5	−	−	−	−	−
**22**	23.6	lobetyolin	C_20_H_28_O_8_	[M + H]^+^ 397.1852 [M + NH_4_]^+^ 414.2062 * [M + Na]^+^ 419.1627	129.1, 155.1, 199.1, 217.1	−1.2	[M − H]^−^ 395.1725 [M + Cl]^−^ 431.1602 [M + HCOO]^−^ 441.1884 *	59.0, 71.0, 89.0, 113.0, 119.0, 143.1, 159.1, 185.1	6.2	[4,5,6]
**23**	24.6	3’-methoxyl-linarin	C_29_H_34_O_15_	[M + H]^+^ 623.1986	315.1, 477.2	2.5	[M − H]^−^ 621.1826 [M + Cl]^−^ 657.1519 [M + HCOO]^−^ 667.1775	−	1.9	[19]
**24**	26.6	linarin	C_28_H_32_O_14_	[M + H]^+^ 593.1868	285.1, 447.1	0.5	[M − H]^−^ 591.1722 [M + Cl]^−^ 627.1536 * [M + HCOO]^−^ 637.1788	268.1, 283.1	2.3	[4,5]
**25**	29.6	diosmetin	C_16_H_12_O_6_	[M + H]^+^ 301.0708	153.0, 229.1, 258.1, 286.1	0.4	[M − H]^−^ 299.0560	284.0	3.3	[20]

* represents the selected precursor ion for MS/MS analysis when multiple pseudo−molecular ions were detected.
